# Pollution from Fossil-Fuel Combustion is the Leading Environmental Threat to Global Pediatric Health and Equity: Solutions Exist

**DOI:** 10.3390/ijerph15010016

**Published:** 2017-12-23

**Authors:** Frederica Perera

**Affiliations:** Columbia Center for Children’s Environmental Health, Department of Environmental Health Sciences, Mailman School of Public Health, Columbia University, 722 W. 168th Street, New York, NY 10032, USA; fpp1@cumc.columbia.edu

**Keywords:** children’s health, fossil fuel emissions, air pollution, climate change, neurodevelopment, benefits of intervention, policy

## Abstract

Fossil-fuel combustion by-products are the world’s most significant threat to children’s health and future and are major contributors to global inequality and environmental injustice. The emissions include a myriad of toxic air pollutants and carbon dioxide (CO_2_), which is the most important human-produced climate-altering greenhouse gas. Synergies between air pollution and climate change can magnify the harm to children. Impacts include impairment of cognitive and behavioral development, respiratory illness, and other chronic diseases—all of which may be “seeded“ in utero and affect health and functioning immediately and over the life course. By impairing children’s health, ability to learn, and potential to contribute to society, pollution and climate change cause children to become less resilient and the communities they live in to become less equitable. The developing fetus and young child are disproportionately affected by these exposures because of their immature defense mechanisms and rapid development, especially those in low- and middle-income countries where poverty and lack of resources compound the effects. No country is spared, however: even high-income countries, especially low-income communities and communities of color within them, are experiencing impacts of fossil fuel-related pollution, climate change and resultant widening inequality and environmental injustice. Global pediatric health is at a tipping point, with catastrophic consequences in the absence of bold action. Fortunately, technologies and interventions are at hand to reduce and prevent pollution and climate change, with large economic benefits documented or predicted. All cultures and communities share a concern for the health and well-being of present and future children: this shared value provides a politically powerful lever for action. The purpose of this commentary is to briefly review the data on the health impacts of fossil-fuel pollution, highlighting the neurodevelopmental impacts, and to briefly describe available means to achieve a low-carbon economy, and some examples of interventions that have benefited health and the economy.

## 1. Introduction

Children, and especially the poor, bear a disproportionate burden of disease and developmental impairment from both environmental pollution and climate change due to the combustion of coal, oil, gasoline, diesel and natural gas. Assessments of the health and economic costs of the impacts of fossil-fuel combustion by-products on children have typically been fragmented, published in specialized journals and have separately considered air pollution and climate change. This silo effect has precluded a full reckoning of the harm to children that results from a carbon-based economy and has stymied the advancement of properly comprehensive policies to protect this vulnerable group. This commentary calls for a holistic accounting of the harm from fossil-fuel burning. Such an accounting is needed to spur the required global mitigation and action to reduce disparities between regions and socioeconomic classes and address the growing threat to future generations. Unless we act forcefully right now, our children and theirs will inherit an unsustainable world, lacking the essential ecological resources and social stability to support them. A major theme of this commentary is environmental injustice: the disproportionately heavy health and economic burden that falls on the young, the poor, and certain minorities, especially those in developing countries who are most vulnerable to the impacts of toxic air pollutants as well as CO_2_-driven climate change resulting from the combustion of fossil fuel. Alleviating this burden would bring great and lasting benefits to children and their progeny. In his Encyclical, *Laudate Si*, Pope Francis concluded that global capitalism, based on the burning of fossil fuels, has created unsustainable consumption and mounting inequity. This warning was also sounded in the recent report of the Lancet Commission on Health and Climate Change [1]. To paraphrase the Lancet Commission: government policies and other strategies to reduce dependence on fossil fuel and build sustainable communities represent the biggest opportunity of our century to improve public health, redress inequality and increase the resilience of individuals, communities and the broader society.

In a prior commentary [2], I envisioned fossil fuel as the modern day version of the many-headed Hydra in Greek mythology, inflicting multiple forms of health and developmental harm in children through its emissions of toxic pollutants and carbon dioxide (CO_2_). Were we to slay the hydra by transitioning to sustainable and renewable energy sources for transport, electricity generation, and industry, we would reap lasting benefits for children. These include significantly fewer cases of babies born preterm or with low birth weight, children with cognitive and behavioral disorders, mental-health problems, asthma and other respiratory illness, and potentially cardiovascular disease and cancer—all of which have been linked to toxic air pollutants. Mitigation of climate change would mean fewer children suffering from heat-related disease, malnutrition, infectious disease, physical and psychological trauma, mental-health problems, and respiratory illness. All of these health benefits would occur immediately and play out over the life course, since exposure-related damage, disease, or impairment in early life can affect long-term health and functioning. Of growing concern are the adverse effects on early brain development, impairing children’s ability to learn, hence their future economic productivity and ability to contribute ideas and energy to society. As a result, they, their families and the broader community are less resilient (“able to survive, adapt, and grow in the face of stress and shocks and transform when conditions require”) [3]. Society becomes even less fair, since the children who are most affected are the poor and disadvantaged.

This commentary builds upon a prior review by this author that contains more detail and references [2], updating it with more recent or additional scientific and economic data on the current and projected health impacts of fossil-fuel combustion. A major focus here is on the neurodevelopmental impacts of air toxics and climate change and their combination, because heretofore the effects on the developing brain have been less recognized than the other health impacts of these exposures.

## 2. The Young Are Especially Vulnerable to Air Pollution and Climate Change

The developing fetus and young child are more biologically and psychologically vulnerable than adults to the many adverse effects of toxic air pollutants and climate change from fossil-fuel combustion. This differential susceptibility is due to their rapid growth, dynamic developmental programming, immature detoxification, immune, and thermoregulatory systems, and their dependence on adult caretakers. The complexity of early development conveys vulnerability to disruption by toxic exposures of all kinds, including toxic pollutants and stress. Like the respiratory and immune systems, the brain continues to develop during infancy through childhood; however, the prenatal period is considered to be the most dynamic. For example, between conception and birth the brain undergoes highly synchronized maturation from the initial formation of the neural tube to the proliferation and migration of the neurons, synaptogenesis, apoptosis or “pruning” of synapses, and the early phase of myelination [4]. Most of the more than 86 billion neurons of the mature brain are formed during the prenatal period [5]. Numerous studies demonstrate that the fetal and early childhood stages are especially vulnerable to both genetic damage and epigenetic dysregulation from exposure to xenobiotics and stress; these molecular effects may have lifelong and transgenerational consequence [6,7,8].

In addition, because children breathe more air per kilogram of body weight than do adults and require three to four times the amount of food on a body-weight basis than adults, they are more exposed to pollutants in air and food [9,10]. Testifying to the differential vulnerability of the young, the World Health Organization (WHO) has estimated that more than 40% of the burden of environmentally related disease and more than 88% of the burden of climate change is borne by children under 5, although that age group constitutes only 10% of the global population [11,12,13]. The most serious impacts of climate change are occurring in developing countries; however, the entire global population is affected.

## 3. Fossil-Fuel Combustion is the Major Source of Global Air Pollution and CO_2_

Globally, the majority of air pollution is generated by the combustion of fossil fuel (coal, diesel fuel, gasoline, oil, and natural gas) for electricity production, heating, transportation, and industry [14]. Worldwide, in 2011, fossil fuels represented 82% of the total primary energy supply [15]. In the US, oil, natural gas, and coal account for 81% of current fuel use [16]. Energy-related fossil-fuel combustion in high- and middle-income countries and biomass burning in low-income countries accounts for most of the global air pollution, generating 85% of airborne respirable particulate pollution and almost all sulfur dioxide and nitrogen oxide emissions to the atmosphere [17]. Also emitted are black carbon, polycyclic aromatic hydrocarbons (PAH), nitrogen and sulfur dioxides, mercury, and volatile chemicals that form ground-level ozone (O_3_). All are associated with multiple adverse health effects in children.

Air pollution affects practically all countries in the world and all parts of society; only one person in 10 lives in a city that complies with the WHO Air quality guidelines [18]. Household air pollution is an important risk factor for an estimated 2.9 billion people worldwide, especially those in low- and middle-income countries where biomass fuels and coal are commonly burned for cooking and heating [19] In total, about 2 billion children live in areas that exceed the WHO annual guideline for fine particles of 10 μg/m^3^ (the concentration of fine particulate matter that constitutes a long-term inhalation hazard) [20]. About 300 million children currently live in areas where outdoor air pollution exceeds international guidelines at least six-fold [21]. These guidelines are likely underestimates, since recent epidemiological studies have reported impacts below these levels.

Globally, although it remains a very important risk, indoor air pollution has been on the decline in recent years due largely to the reduction in use of solid fuels for cooking from around 60% of homes in 1980 to 42% in 2012 [22]. In contrast, ambient air pollution has shown a dramatic rise. Urban ambient air pollution increased by about 8% between 2008 and 2013; and the upward trend is projected to continue [21].

Fossil-fuel combustion is also the major human source of the greenhouse gases and short-lived climate pollutants that drive climate change. As stated in a recent US interagency report: “Many lines of evidence demonstrate that human activities, especially emissions of greenhouse gases, are primarily responsible for the observed climate changes in the industrial era, especially over the last six decades” [23]. Human activities emit about 35 billion metric tons of carbon dioxide into the atmosphere every year, primarily from energy use [24]. The Annual Greenhouse Gas Index, which is used by the National Oceanic and Atmospheric Administration (NOAA) to track the warming influence of long-lived climate-altering greenhouse gases, increased by 40% from 1990 to 2016, with most of that increase attributable to rising CO_2_ levels [25]. CO_2_ levels in the atmosphere are at their highest in 800,000 years [26]. In the US, coal and natural gas are the largest contributors to carbon pollution (constituting a third of all domestic carbon emissions). Methane released by production of natural gas, oil, and coal is second in importance. Although natural gas emits significantly less toxic air pollution and CO_2_ than the other fossil fuels [27], the drilling, extraction, and transportation of natural gas results in the leakage of methane that is 34 times more effective than CO_2_ at trapping heat over a 100-year period [28]. Given its increasing share of total fuel use, natural gas is expected to surpass coal as a source of energy-related CO_2_ emissions in the US [29].

According to the International Energy Agency, worldwide growth in coal consumption is predicted to decline between 2015 and 2021 as developed countries continue to abandon coal as an energy source and China’s consumption plateaus. However, that decline will be offset by growing demand among emerging nations, particularly in India and south-east Asia. Based on the assumption of business as usual, the US Energy Information Administration predicts that coal will remain the second-largest energy source worldwide, following petroleum and other liquid fuels, until 2030; and from 2030 through 2040, it will be the third-largest energy source, surpassed only by liquid fuels and natural gas [30]. In the US and some other countries, carbon emissions from cars and trucks have exceeded carbon emissions from electric power [31].

## 4. Fossil-Fuel Combustion-Related Air Pollutants and Climate Change are a Major Cause of Environmental Injustice

A major environmental injustice is that children, who are dependent on adults and did not create the problems, bear the brunt of the impacts of air pollution and climate change. It is a further injustice that children in low- and middle-income countries as well as lower-income communities and communities of color in high-income countries like the US are disproportionately affected. Echoing an earlier WHO finding, the recent report of the Lancet Commission on Pollution and Health, on which this author was a commissioner, stated that pollution in all its forms disproportionately affects the poor and marginalized in every country worldwide, with air pollution being the largest contributor to pollution-related deaths, mainly in low- and middle-income countries [32]. Low- and middle-income countries in the WHO South-East Asia and Western Pacific Regions had the greatest air pollution-related burden in 2012, with a total of 2.6 million deaths related to outdoor air pollution and 3.3 million deaths linked to indoor air pollution [33]. However, in contrast to pollution-related deaths, which largely occur among adults over 60, disability-adjusted life years (DALYs) resulting from pollution-related disease are highly concentrated among infants and young children, reflecting the vulnerability of the young and the many years of life lost with each death of a child [34].

Two factors contribute to the socioeconomic disparities in the impacts of air pollution: differential exposure and heightened susceptibility. Globally, there is a notable pattern of disproportionate exposure of the poor and of certain racial/ethnic groups to air pollution. Studies have shown that low-income communities and communities of color in the US experience disproportionately high exposure to particulate air pollution and air pollution from coal-fired power plants [2]. A GIS-based study of over 150,000 children in the US found that the distribution of three stationary and mobile air-pollution sources followed a consistent pattern of racial inequity, with Hispanic and black children facing significantly higher levels of potential exposure than white children [35]. With respect to heightened susceptibility, an analysis of the Mexico City scenario concluded that low socioeconomic status (SES) children in that megacity are not only exposed to high levels of pollution buttend to have inadequate nutrition and deficient schools, and often face domestic, school and street violence . Because these co-factors make children more vulnerable to air toxins, the significant impact of high air pollution is likely affecting predominantly low SES children in Mexico City [36].

The same factors contribute to disparities in the impacts of climate change. It is the poor who are most often forced to live in areas that are especially vulnerable to extreme flooding, drought and other impacts of climate change. Poor children are also less buffered and less resilient in the face of climate change: “a child living in poverty or deprived of adequate water and sanitation before a crisis will be more affected by a flood, drought or storm, less likely to recover quickly and at even greater risk in a subsequent crisis” [37]. Pre-existing inadequate nutrition, lack of adequate social support, and psychosocial stress due to poverty magnify the effects of both climate change and air pollution. Worldwide, the number of children living in poverty is staggering: one billion children, almost half of the 2.2 billion children below 15 years of age, are living in poverty [38]. In the US, the world’s most prosperous country, the child poverty rate is a shocking 22%. As will be discussed, our research following parallel birth cohorts in the US and Poland has found that co-exposure of pregnant women to air pollution and social stress or hardship due to poverty significantly increases the adverse effect of air pollution on children’s IQ and behavioral problems including attention deficit hyperactivity disorder (ADHD) [39,40,41]. For example, our research in Poland found evidence of significant interactions between maternal demoralization during pregnancy, itself correlated with material hardship, and prenatal air-pollution exposure on children’s behavioral problems [41]. In our NYC research, combined prenatal exposure to PAH and material hardship was associated with a significant reduction in the IQ of children [39].

## 5. The Health Impacts of Air Pollution in Children Include Mortality and Neurodevelopmental Problems

The Lancet Commission on Pollution and Health [32] noted that air pollution remains one of the great killers of our age, echoing an earlier conclusion by the WHO that: “Air pollution, both ambient (outdoor) and household (indoor), is a public health emergency” and the biggest environmental risk to health. Most of the attention has been on excess mortality in the overall population, with the largest number of deaths in adults [33,42]. However, in children under 5 years’ old, 1.7 million deaths are attributed to pollution and environmental risks in general; with air pollution linked to 600,000 of these deaths each year, largely due to pneumonia [37,43]. Because of the increasing trend in outdoor air-pollution levels, according to the Organization for Economic Co-operation and Development (OECD), under-5 mortality could be 50% higher than—or even double—current estimates by 2050 as a result of outdoor air pollution [22]. It is predicted that by 2050 outdoor air pollution will become the leading cause of child death [37]. However, millions more children are affected by chronic illness, including respiratory illness other than pneumonia and effects on physical and cognitive development [37].

A previous commentary by this author reviewed in some detail the documented effects of combustion-related air pollution on multiple outcomes including adverse birth outcomes, respiratory illness, and cancer [2]. Here the focus is on neurodevelopmental impacts, an area that has been under-recognized. Low birth weight and preterm birth will be briefly discussed in the context of child neurodevelopment.

A growing body of evidence indicates that early-life exposure to combustion-related air pollutants adversely affects children’s cognitive and behavioral development. There are inconsistencies in the results among studies that can be explained in part by differences in measures used and levels of air pollution, in race/ethnicity, potentially in susceptibility, and in methods to assess children’s neurodevelopment.

Particulate matter (PM) and traffic-related pollutants: Many studies have assessed the associations between PM or traffic-related air pollutants and cognitive outcomes in children from cohorts in the US, Europe and Asia [44,45,46,47,48,49,50,51,52,53,54]. The studies relied on estimates of exposure to PM or traffic-related pollutants (black carbon or nitrogen dioxide/NO_2_) largely based on land-use regression models, distance of the maternal residence during pregnancy to roadways, or traffic density. Although the results for PM have been mixed, most of the studies on traffic-related pollutant exposure have reported associations with decreased mental and psychomotor development. Several studies found that the two traffic-related pollutants were associated with reductions in children’s memory and IQ, after sociodemographic factors were taken into account [52,53]. Exposure to traffic pollution in childhood has also been linked to slower brain maturation [55].

With respect to attention problems, a study in Boston found more such problems with higher exposure to fine, respirable particulate matter having an aerodynamic diameter of less than 2.5 microns (PM_2.5_), during the sensitive periods of gestation, with differences between boys and girls [56]. Studies in South Korea and Japan also reported more attentional problems with higher prenatal exposure to PM or other pollutants [57,58]. The association with prenatal exposure to traffic-related air pollutants was not observed in German cohort studies; but there was a significant association between higher PM_2.5_ levels at the children’s current addresses and increased hyperactivity/inattention scores [45].

Air pollution, mainly PM_2.5_ or traffic-related pollution, has been associated in a number of studies with autism spectrum disorder (ASD) [59,60,61,62,63]. In contrast, European studies reported no association with autistic traits [64,65]. Although studies have not all been consistent, there is growing evidence that prenatal exposure to traffic-related air pollutants and PM_2.5_ may be risk factors for ASD [66,67,68].

There is some evidence using molecular biomarkers and magnetic resonance imaging (MRI) of the brain that chronic exposure to airborne pollutants in the early years may contribute to neurodegenerative disease processes including Alzheimers’s disease [69,70]. In Mexico City, researchers have reported distinct brain changes that have been associated with adult neurodegenerative disease in children living in high air-pollution areas [69].

Polycyclic aromatic hydrocarbons (PAH): PAH are a class of neurotoxic air pollutants that my colleagues and I have studied with respect to cognitive and behavioral outcomes and mood disorders in complementary cohort studies in New York City, Krakow, Poland and Chongqing, China [39,71,72,73,74,75,76,77,78,79,80,81,82,83,84,85,86,87,88] In our NYC cohort, prenatal exposure to PAH measured by personal air-monitoring or biomarkers of PAH exposure in cord or maternal blood was associated with developmental delay, reduced IQ, symptoms of anxiety, depression, and inattention, ADHD, deficient maturation of emotional self–regulation capacity, and poorer social responsiveness in childhood. Significant interactions were observed between prenatal PAH and material hardship due to poverty on child IQ [39] and between prenatal PAH and maternal psychological distress on mood-related problems [2]. More recently, significant combined effects of PAH and material hardship have been observed on ADHD outcomes [40]. MRI brain imaging of a subset of children in the NYC cohort showed significant correlations between measures of prenatal PAH exposure and distinct anatomical changes [80].

An example of the neurodevelopmental benefits of reducing air-pollution levels is provided by our research in Tongliang, China, that compared a cohort born before the closure of a centrally located coal power plant to a cohort conceived after plant closure. The second cohort had more favorable birth and neurodevelopmental outcomes, significantly lower cord blood levels of the biomarker of exposure measured in cord blood (PAH-DNA adducts), higher levels of a protein important in early brain development known as brain-derived neurotrophic factor (BDNF), and longer telomeres, a general marker of health [89,90].

In addition to their immediate toll, preterm birth and low birth weight are known risk factors for a number of neurodevelopmental disorders in children [91]. Recent studies confirm the reproductive effects of air pollution, their socioeconomic and racial disparities and the large cost imposed on affected families, furthering socioeconomic inequality and increasing the risk of neurodevelopmental effects in vulnerable populations. For example, a large prospective study in China found a significant increase in preterm birth with each 5 µg/m^3^ increase in maternal exposure to PM_2.5_ during the pregnancy [92]. A recent analysis found that about 2.7 million premature births per year (18% of preterm births) globally are associated with PM_2.5_ exposure, including from fossil-fuel and biomass burning, mostly in developing countries [93]. A recent multi-country study concluded that, across all study populations, maternal exposure to particulate pollution was associated with low birth weight at term [94]. Racial disparities exist. In the US, preterm birth rates are 7.4% among Non-Hispanic white infants compared to 17.2% for Non-Hispanic black infants. Both social and physical environmental factors contribute to these disparities [95]. These effects are costly to society and individuals. In the US alone, PM_2.5_ caused an estimated 15,000 preterm births in 2010, costing about $5 billion in medical care, special education services and lost economic productivity for that single year’s cohort [96].

## 6. Climate Change is Linked to Serious Health Impacts in Children Including Mortality and Developmental Impairment

As noted in the Introduction, references for this section can be found in [2]. Increased illness, injury, and deaths from heat stress, floods, drought, and increased frequency of forest fires and intense storms are among the direct effects of climate change. The indirect effects include malnutrition and under-nutrition, the spread of infectious-disease vectors, food insecurity, illness due to increased air pollution and aeroallergens, and mental ill health from displacement, social and political instability. Children are especially vulnerable to both the indirect and direct consequences of climate change.

There is broad scientific agreement that climate change has already taken a significant toll on children and that the impacts will increase dramatically unless forceful action is taken. The WHO estimated that climate change since the mid-1970s contributed to about 5 million lost DALYs world-wide in 2000 through malnutrition, diarrhea, and malaria, mostly in children and in developing countries. The toll is expected to rise to 175 million children affected each year in the next several years. These numbers are substantial underestimates since they reflect only a few of the health effects from climate change.

Children bearing the greatest burden of climate-sensitive diseases are those living in regions with the least capacity to adapt to risks—regions that have contributed the least in terms of global emissions of greenhouse gases. Although children in developing countries bear the brunt, the impacts of climate change are increasingly being seen in the US and Europe, especially among populations of low socioeconomic status.

Virtually all of the impacts of climate change can affect children’s neurodevelopment, cognitive functioning, behavior, and mental health, either directly or indirectly. Further, as with air pollution, the impacts are likely to be felt over the lifetime, affecting resilience, health and productivity. Climate change impacts the development of children’s brains in many ways. Malnutrition during the first 1000 days causes stunting of the brain and body, with associated reduced neurodevelopmental and cognitive function in children and subsequent decreased ability to learn and be economically productive [97]. In 2017, 155 million children under five (1 in 4 children) are stunted due to hunger [98].

Stress from extreme weather events also contributes to neurodevelopmental and mental health problems in children. Although no single extreme weather event, such as floods, droughts, wildfires, or hurricanes and cyclones, can be attributed directly to climate change, human-induced climate change is contributing to the frequency and severity of such events. Over the last several decades, there have also been more intense and frequent heat waves as well as marked regional changes in floods, droughts and wildfires in certain parts of the US and globally [99]. An estimated 66.5 million children world-wide were directly affected by weather-related disasters every year from 1990–2000, of whom 600,000 died. Sea-level rise due to global climate warming has made coastal storms increasingly dangerous for coastal infrastructure and inhabitants, contributing to deaths from drowning. Drowning is a major cause of fatality in children in developing countries. According to a recent study, rates of sea-level rise between 1993 and 2011 exceeded by 60% the highest projections made in 2007 by the Intergovernmental Panel on Climate Change. In 2017, the NOAA estimated that the sea-level rise by the end of this century could reach as high as 6.5 feet, enough to inundate many waterfront cities around the globe [100]. Notable extreme weather events in the last decade include the massive flooding across south-east Asia in 2011 which affected an estimated 9.6 million people, many of them children [101]. In the US, Hurricane Katrina in 2005 forced 1 million people in New Orleans from their homes and left 372,000 children without schools; and Hurricane Sandy in 2012 affected people in 8 countries including 24 states in the US, with particularly severe damage in New Jersey and New York. Children who were affected by Hurricane Katrina were found to have higher rates of anxiety and depression [102]. Most recently, in September 2017 Hurricane Irma devastated islands in the Caribbean; and massive flooding in South Asia placed almost 16 million children in urgent need of life-saving support [103]. While populations of all economic statuses have been impacted, these events have most seriously affected the children in low-income communities.

The psychological and emotional impacts of climate change include the acute, traumatic effects of extreme weather events, mental and emotional distress resulting from direct experience or anxiety about future risks, chronic stress from heat, drought, forced migrations, and climate-related conflicts, and the stress of adjustment in the wake of weather-related disasters. Migration and population displacement as a result of social and political instability due to climate change affects the mental health of children in low-income, developing countries, contributing to the perpetuation of poverty and civil unrest. These countries, in which children less than 18 years’ old represent 50% of their population, already bear most of the global burden of poverty and childhood disease.

## 7. Prenatal or Childhood Exposure to Air Toxics and Climate Change Can Have Long-Term and Synergistic or Combined Health Impacts

By launching a trajectory of adverse effects following the initial physical or developmental impairment, and/or by “seeding” latent disease that only becomes evident in later life, toxic air pollutants can affect health and functioning over the life-course. For example, adverse reproductive outcomes associated with *in utero* environmental exposures are risk factors for neurodevelopmental, respiratory, and other health problems in infancy and childhood, as well as heart disease, chronic obstructive pulmonary disease, and diabetes in adulthood. As noted above, childhood ADHD and ASD have been associated with early-life exposure to air pollution; these disorders may persist into adulthood, affecting professional and personal life and increasing the costs of healthcare for individuals and families. There is empirical evidence that early childhood exposure to air pollution is linked to lower scores on IQ and other intelligence tests, with long-term economic consequences in terms of lifetime earnings.

Similarly, the impacts of climate change can also play out over a child’s lifetime. As noted, stunting of children’s bodies and brains due to malnutrition during the first 1000 days results in impairment of cognitive functioning and learning. Early adversity and toxic stress are also linked to impairments in learning, behavior, and physical and mental health.

There is an increasing body of evidence that early-life exposures to air pollutants, nutritional deprivation, and stress can result in transgenerational impacts, possibly via the transmission of epigenetic changes. Combustion-related PAH have been shown to alter epigenetic marks in newborns, potentially dysregulating genes involved in disease pathways.

Although such effects have not been adequately documented, there can be harmful synergy or combined effects of toxic air emissions from the burning of fossil fuels and climate change. In California, during the month of November 2017 toxic air pollution from 22 concurrent fires has affected millions of people, adding to the pollution from traffic and stationary sources [104]. As another example, the effect of air-pollution exposure during pregnancy on risk of preterm birth is likely to be magnified by concurrent experience of extreme temperatures, food insecurity and stress due to climate change. The same is true for effects of co-exposure to air toxics, malnutrition and stress on child neurodevelopment. Children affected by malnutrition are likely to be more vulnerable to the neurotoxic effects of air pollution; and children born with low birth weight or preterm due to air pollution will be at greater risk of malnutrition or infectious disease.

## 8. Economic Benefits of Action are Underestimated but Significant

Lacking in the literature and in the public understanding is a holistic assessment of the economic costs of the many impacts of fossil-fuel combustion on children’s health, hence the full economic benefits of action to reduce or prevent these impacts. The numbers below do not capture the full costs to individuals, families and society in terms of direct medical costs, costs to healthcare systems, opportunity costs resulting from lost productivity, and lower economic growth. However, even the limited available estimates of the monetary costs of deaths and morbidity from air pollution or climate change, individually, are staggering. The available data indicate that reduction of dependence on fossil fuel will bring very large economic benefits. They demonstrate the false dichotomy between regulation of air pollution and economic growth. Estimates of the avoided and avoidable economic health costs of air pollution and climate change include the following:

- Avoided health costs attributed to the US Clean Air amendments: ~$2 trillion for the year 2020 [105];
- A prediction of ~$250 billion/year by 2030 ($140 billion to $1050 billion) in avoided health costs from clean energy policies in the US [106];
- An estimated $361 to $886 billion/year in health costs due to US fossil fuel electricity [105,107];
- An estimated cost of ~$187 billion/year due to air pollution from coal combustion in the US [108];
- Gains in lifetime earnings related to a hypothetical 25% reduction in PAH in NYC air: $215 million for each annual NYC birth cohort of Medicaid births [109];
- An estimate of $3.5 trillion/year in costs of ambient air pollution in OECD countries, India and China [1];
- Total annual costs of air pollution currently estimated to be approximately 0.3 per cent of global GDP and expected to increase to approximately 1 per cent of GDP by 2060 [110];
- Reductions in airborne particulate matter between 2001 and 2010 in Taiyuan, Shanxi province, China associated with 2810 fewer premature deaths, 31,810 fewer hospital admissions, 141,457 fewer outpatient visits, 969 fewer emergency department visits, 951 fewer cases of bronchitis and more than 30,000 fewer DALYs attributed to air pollution in Taiyuan in 2010 compared to 2001. The decrease in the estimated cost of premature death due to air pollution: 3.83 billion Yuan, or approximately $621 million USD [111];
- Estimated cost of deaths from air pollution to the global economy: about $225 billion in lost labor income and more than $5 trillion in welfare losses in 2013 (World Bank/Institute for Health Metrics and Evaluation study, cited in WHO, Clear the Air) [112];
- Estimated ~$14 billion cost due to six climate-change-related events in the US between 2002 and 2009 [113];
- The expected benefits of the California climate change program: a $76 billion increase in the state’s Gross State Product, a $48 billion increase in real household incomes, and the creation of 403,000 new efficiency- and climate-driven jobs [114];
- Between 1980 and 2017, the cost of 208 extreme weather and climate events in the US: at least $1 billion each, with total damages of more than $1.1 trillion, and a similar increase in these costly events happening around the world [115];
- The estimated global cost of climate change from deaths and diseases such as diarrhea, malnutrition, malaria, and heat stress up to $4 billion per year by 2030 [116];
- Globally, up to $230 billion of avoided external health costs each year by 2030 with an increase to 36% renewables in global energy consumption by 2030 [1].

## 9. Solutions Exist and Interventions are Being Mounted

Means are at hand, and already at work in many communities, cities and countries to transition from dirty fossil fuels to clean energy. This transition is seen by experts as both the major challenge and the major opportunity of our time [1]. Expert groups have underscored the feasibility of seizing this opportunity, citing policies and initiatives that have been effective. They have also underscored that to be successful and benefit the health and future well-being of children worldwide, the transition from dirty fossil fuels to clean energy must be done equitably and inclusive of all communities, especially those that are disadvantaged [117].

The WHO [42] describes a number of policies in transport, urban planning, power generation and industry that are known to be effective in reducing emissions of fossil fuel-related air toxics and CO_2_:

- Clean technologies that reduce industrial smokestack emissions; improved management of urban and agricultural waste, including the capture of methane gas emitted from waste sites as an alternative to incineration (for use as biogas);
- Shifting to clean modes of power generation; prioritizing rapid urban transit, walking and cycling networks in cities as well as rail inter-urban freight and passenger travel; shifting from heavy-duty diesel vehicles to low-emission vehicles and fuels, including fuels with reduced sulfur content;
- Improving the energy efficiency of buildings and making cities more compact, and thus energy efficient;
- Increased use of low-emissions fuels and renewable combustion-free power sources (like solar, wind or hydropower); co-generation of heat and power; and distributed energy generation (e.g., mini-grids and rooftop solar-power generation).

According to the International Energy Agency (IEA), “the technologies for the energy sector to push air pollution levels into a steep decline in all countries exist and are in widespread use today; and they can be applied at great net economic benefit. Such actions can help avoid millions of pollution-related deaths; greenhouse-gas emissions would also be cut and fossil-fuel import bills reduced” [17]. In fact, many countries have lowered CO_2_ emissions through fuel-economy standards for auto emissions, limits for power plants, stricter energy-efficiency codes for buildings, and other available methods.

Examples of interventions now being mounted to address climate change and fossil fuel-related pollution include the India Heat Action Plan (HAP) [118]. The HAP has provided an early-warning system to better prepare and protect local communities from deadly heat waves. Along with the immediate benefit in terms of adaptation to the current threat, the increased awareness of climate change is incentivizing the Indian government to move away from coal and other fossil fuel.

Another is the California climate change initiative launched in 2006 as a multi-year program to reduce greenhouse-gas emissions in California, with a 2030 greenhouse-gas emissions reduction target of 40% below 1990 levels. The expected benefits included a $76 billion increase in the state’s Gross State Product, a $48 billion increase in real household incomes, the creation of over 400,000 new efficiency- and climate-driven jobs, and more than $8 billion by 2025 in pollution-related health costs avoided [114]. With respect to equity, the program is designed to ensure that the benefits of energy efficiency reach low-income residents as well as middle- and high-income residents.

The regional initiative to reduce air pollution and CO_2_ emissions in the north-eastern states (US) known as the Regional Greenhouse Gas Initiative (RGGI) placed a regional limit on the amount of CO_2_ that power plants can emit and instituted a cap-and-trade policy. A 2017 analysis found that RGGI created major benefits to public health and productivity including the avoidance of 300–830 early deaths among adults; 39,000–47,000 lost work days; and 35–390 non-fatal heart attacks [119]. The total health-cost savings from RGGI to date are estimated to be $5.7 billion.

Another example is the provincial-level initiative in Taiyuan, Shanxi Province, China. Shanxi Province is the major coal-mining and coal-burning region. Taiyuan, the capital of Shanxi Province, has, in the past, been counted as one of the world’s worst cities for air quality. The Columbia Center for Children’s Environmental Health (CCCEH) at the Mailman School of Public Health, the Shanxi Medical University, the Center of Disease Control and Prevention of Taiyuan Municipality, and Shanghai Fudan University School of Public Health estimated the health and economic benefits of policies between 2001–2010 to reduce the burden of air pollution in Taiyuan [81]. They include 30,000 fewer DALYs attributed to air pollution in Taiyuan in 2010 compared to 2001 and economic savings in avoided health costs of premature death due to air pollution of 3.83 billion Yuan, or approximately $621 million. The team is now updating these findings, assessing subsequent policy changes from 2010 to 2016, including additional health outcomes in children, and incorporating additional satellite and ground-level monitoring data.

A final example is the Paris Agreement [120]. Unfortunately, alone among the 195 signers of the Treaty, the current US Administration has stated the intention to withdraw from this major international treaty. The US Environmental Protection Agency (EPA) report, Climate Change in the United States: Benefits of Global Action [121], estimated that billions of dollars of avoided damages in the US would result from global efforts to reduce greenhouse-gas emissions. These included a significant portion of the health costs attributed to air pollution and climate change. The Clean Power Plan, now being rolled back by the current administration, was intended to play a key role in meeting the targets set by the Paris Agreement. From a benefit-cost perspective, the EPA estimated that the air pollution co-benefits of the Clean Power Plan were worth $25–$62 billion, far more than the estimated $7–$9 billion in compliance costs [122]. Adding in global climate benefits increased total benefits to $55–$93 billion.

These examples are encouraging but also pose the challenge of more fully documenting their health and economic benefits and the extent to which we promote environmental justice. Fortunately, methods are in place to make advances in that area and efforts are ongoing.

## 10. Conclusions

Consideration of the full impacts of fossil-fuel pollution and our carbon-based economy shows that unless strong action is taken now, our children and their progeny will inherit an increasingly unsustainable and unfair world in which they, their families and communities will not be able to survive, adapt, grow and transform where needed. The mounting health and economic costs of pollution and climate change from fossil-fuel combustion are already spurring mitigation efforts that can serve as models for other communities, and regional, state and global entities. These provide hope for the future.
